# Speech Features as Predictors of Momentary Depression Severity in Patients With Depressive Disorder Undergoing Sleep Deprivation Therapy: Ambulatory Assessment Pilot Study

**DOI:** 10.2196/49222

**Published:** 2024-01-18

**Authors:** Lisa-Marie Wadle, Ulrich W Ebner-Priemer, Jerome C Foo, Yoshiharu Yamamoto, Fabian Streit, Stephanie H Witt, Josef Frank, Lea Zillich, Matthias F Limberger, Ayimnisagul Ablimit, Tanja Schultz, Maria Gilles, Marcella Rietschel, Lea Sirignano

**Affiliations:** 1 Mental mHealth Lab Institute of Sports and Sports Science Karlsruhe Institute of Technology Karlsruhe Germany; 2 Department of Psychiatry and Psychotherapy Central Institute of Mental Health University of Heidelberg Mannheim Germany; 3 Department of Genetic Epidemiology in Psychiatry Central Institute of Mental Health University of Heidelberg Mannheim Germany; 4 Institute for Psychopharmacology Central Institute of Mental Health University of Heidelberg Mannheim Germany; 5 Department of Psychiatry College of Health Sciences University of Alberta Edmonton, AB Canada; 6 Educational Physiology Laboratory Graduate School of Education University of Tokyo Tokyo Japan; 7 Cognitive Systems Lab University of Bremen Bremen Germany

**Keywords:** ambulatory assessment, experience sampling, ecological momentary assessment, speech features, speech pattern, depression, sleep deprivation therapy, mobile phone

## Abstract

**Background:**

The use of mobile devices to continuously monitor objectively extracted parameters of depressive symptomatology is seen as an important step in the understanding and prevention of upcoming depressive episodes. Speech features such as pitch variability, speech pauses, and speech rate are promising indicators, but empirical evidence is limited, given the variability of study designs.

**Objective:**

Previous research studies have found different speech patterns when comparing single speech recordings between patients and healthy controls, but only a few studies have used repeated assessments to compare depressive and nondepressive episodes within the same patient. To our knowledge, no study has used a series of measurements within patients with depression (eg, intensive longitudinal data) to model the dynamic ebb and flow of subjectively reported depression and concomitant speech samples. However, such data are indispensable for detecting and ultimately preventing upcoming episodes.

**Methods:**

In this study, we captured voice samples and momentary affect ratings over the course of 3 weeks in a sample of patients (N=30) with an acute depressive episode receiving stationary care. Patients underwent sleep deprivation therapy, a chronotherapeutic intervention that can rapidly improve depression symptomatology. We hypothesized that within-person variability in depressive and affective momentary states would be reflected in the following 3 speech features: pitch variability, speech pauses, and speech rate. We parametrized them using the extended Geneva Minimalistic Acoustic Parameter Set (eGeMAPS) from open-source Speech and Music Interpretation by Large-Space Extraction (openSMILE; audEERING GmbH) and extracted them from a transcript. We analyzed the speech features along with self-reported momentary affect ratings, using multilevel linear regression analysis. We analyzed an average of 32 (SD 19.83) assessments per patient.

**Results:**

Analyses revealed that pitch variability, speech pauses, and speech rate were associated with depression severity, positive affect, valence, and energetic arousal; furthermore, speech pauses and speech rate were associated with negative affect, and speech pauses were additionally associated with calmness. Specifically, pitch variability was negatively associated with improved momentary states (ie, lower pitch variability was linked to lower depression severity as well as higher positive affect, valence, and energetic arousal). Speech pauses were negatively associated with improved momentary states, whereas speech rate was positively associated with improved momentary states.

**Conclusions:**

Pitch variability, speech pauses, and speech rate are promising features for the development of clinical prediction technologies to improve patient care as well as timely diagnosis and monitoring of treatment response. Our research is a step forward on the path to developing an automated depression monitoring system, facilitating individually tailored treatments and increased patient empowerment.

## Introduction

### Background

Depression is one of the most prevalent health disorders worldwide [1,2]. The World Health Organization predicted that depression would be 1 of the 3 leading causes of disease burden by 2030 [3], even before its prevalence increased owing to the COVID-19 pandemic [4]. This disorder has symptoms that include depressed mood, loss of energy and interest, sleep problems, and diminished ability to concentrate [5]; thus, depression imposes a substantial burden on the patients as well as their surroundings, society, and the economy [6]. Most importantly, depression is a chronic disorder, characterized by multiple episodes over years or decades. However, strategies for secondary prevention or early detection of new episodes are missing.

The diagnosis and severity assessment of depression relies mostly on self- or caregiver reports, which are prone to retrospective and social desirability bias [7,8]. In addition, such assessments are time and resource intensive because clinical specialists are needed over the course of treatment and recovery [9]. Moreover, many new episodes remain undiagnosed or untreated, that is, secondary prevention is the main issue [10,11]. To reduce burden, the timely detection and diagnosis of (new) depressive episodes are critical.

In recent years, research has focused on the identification of mental health disorder indicators that can be derived automatically, driven by technological developments [12,13]. In particular, the innovation of the ambulatory assessment research technique has contributed strongly to this endeavor [14]. Different terms have been used for this kind of methodology: *ambulatory assessment* [15], *ecological momentary assessment* [16], *experience sampling* [17], and *digital phenotyping* [18]. Although the terms differ, all approaches use computer-assisted methodology to assess momentary self-reported symptoms (eg, via electronic diaries [ediaries]), behaviors, or physiological processes, or actively or passively collect smartphone and physical data or context information (eg, via wearables) while the participant performs normal daily activities in their natural environment [19]. The main advantages of ambulatory assessment are (1) the ability to collect real-life data in real time, thereby reducing retrospective recall bias and increasing ecological validity; and (2) the ability to collect data continuously (passively), which allows us to capture dynamic changes. Accordingly, ambulatory assessment is a promising tool for the timely detection of upcoming clinical episodes to prevent further clinical deterioration [20-22]. In particular, parameters captured objectively by wearables are useful because they can be assessed passively with a high frequency over prolonged time periods [23].

Promising markers that can be assessed objectively are speech and language, which are also metaphorically called “the mirror of the soul” [24]. Even before objective measurements with ambulatory assessment technology were feasible, clinical observations described the voice of patients with depression as low, slow, and hesitant, with these patients speaking in a monotonous and expressionless manner [24,25]. Voice and speech production may be affected by typical characteristics of the clinical nature of depression; for example, psychomotor retardation, energy loss, and cognitive difficulties also affect the vocal folds, leading to a lower *intensity*, *rate,* and *loudness* of speech, which manifest in a monotone and toneless voice [26-28]. Recent reviews have highlighted the potential of using speech markers to assess a variety of psychiatric disorders [29], especially depression [30]. The use of speech as a marker has several advantages because it can be recorded (1) casually; (2) in a noninvasive manner at people’s homes or in public places (with consent provided); and (3) at low cost because microphones are integrated in many devices such as smartphones, smartwatches, and hearing aids. With the availability of open-source speech analysis software (eg, open-source Speech and Music Interpretation by Large-Space Extraction [openSMILE; audEERING GmbH] and Praat) and advances in automatic speech processing technologies based on machine learning techniques, research and development on the use of acoustic and linguistic features to identify mood disorders in particular [29] have been made possible.

### Prior Work

Many studies have successfully discriminated between healthy controls and patients with depression based on speech features [30]. However, understanding within-person (vs between-person) depression-related voice changes is essential in detecting new episodes, that is, the secondary prevention. To the best of our knowledge, only a few studies in samples with clinical (not subclinical) depression have examined the variability of speech features within persons [31-36]. In a 6-week treatment-monitoring study, weekly speech samples were obtained from 35 patients with depression using an interactive voice response system [31]. Patients with an improvement in depressive symptoms showed a significant increase in *pitch* and *pitch variability*, an increase in *speech rate*, and shorter *speech pauses* while speaking at their final assessment compared with their baseline assessment. Importantly, patients whose depressive symptoms did not improve did not show these changes.

The data set of Mundt et al [31] was reanalyzed multiple times [32,34,35]. Quatieri and Malyska [34] integrated additional speech features and identified that lower *pitch variability*, *shimmer*, and *jitter* as well as an increased *harmonics-to-noise ratio* were correlated with lower depression severity. This is in contrast to the study by Mundt et al [31], who found that increased *pitch variability* was associated with lower depression severity, which Quatieri and Malyska [34] attributed to differences in the set of voice samples analyzed (read speech in the study by Mundt et al [31] and conversational speech in the study by Quatieri and Malyska [34] from the same patients).

Trevino et al [32] discussed *speech rate* extraction methods based on the data set of Mundt et al [31] and replicated results regarding *speech rate* in automatically derived phonologically based features. *Speech rate* was negatively correlated with depression scores and the psychomotor retardation item in particular. Moreover, the authors replicated the finding that *speech pauses* were positively correlated with depression severity.

Furthermore, Horwitz et al [35] reanalyzed a subset of data from the study by Mundt et al [31] with a focus on disentangling how speech features relate to the total assessment score and individual symptom items. The authors found a positive correlation between *pitch variability* and depression scores and a slower *speech rate* with increasing depression severity. Notably, they analyzed a different speech task and a different depression assessment in comparison with Mundt et al [31].

Mundt et al [33] replicated their results from Mundt et al [31] in a larger study. Here, 105 patients were observed in a 4-week randomized placebo-controlled study. Again, analyses entailed a comparison of the final and baseline assessments. For patients benefiting from the treatment, *total pause time* was lower, *pitch* was higher (*pitch variability* was not assessed), and *speech rate* was higher. For patients who did not benefit from the treatment, only *speech rate* increased; however, it increased significantly less than in patients benefiting from the treatment.

Yang et al [36] analyzed clinical interviews recorded in 7-week intervals. In contrast to Mundt et al [31], they did not find a change in *pitch variability* with a change in depression severity in the patients but rather in the interviewers. The authors also found shorter *switching pauses* between patient and interviewer (ie, both interlocutors) with lower depression severity.

Although not completely consistent, these findings support the assumption that voice features change within individuals when depression severity changes. However, although data were collected at multiple time points during the study (eg, weekly), except in the study by Yang et al [36], the analysis was limited to a comparison between the baseline and final assessments. However, given that the goal is to detect and ultimately prevent new depressive episodes and deterioration, it is essential to understand within-person trajectories of voice features and how they are associated with momentary states with increased granularity. In this study, we used a naturalistic data set where a rapidly acting antidepressant treatment (ie, sleep deprivation therapy [SDT] [37]) was applied to patients experiencing a depressive episode. The antidepressant effect vanishes in most of the cases after recovery sleep. Baseline, the treatment effect of SDT, and relapse can be measured in a matter of 4 days, making it a preferable setting to study within-person fluctuations.

### Aims and Hypotheses

To investigate the within-person relationship between fluctuations in depression severity and fluctuations in speech features, we used a longitudinal data set with an average of 32 (SD 19.83) assessments per patient. All patients had experienced an acute depressive episode and undergone SDT [37], a chronotherapeutic intervention that can rapidly improve depression symptomatology. The main advantage of this therapeutic is that we maximize the variance of affective states within the data set and ensure sufficient within-person fluctuations over time. As the amount of speech features is immense, resulting in alpha error inflation, we focused on 3 speech features with high face validity that have shown first hints in past research [31-36]. Specifically, we hypothesized that (1) changes in *pitch variability*, (2) shorter *speech pauses*, and (3) higher *speech rate* are associated with lower depression severity. In addition, we assessed the associations of these features with additional momentary affective states (ie, positive affect, negative affect, valence, energetic arousal, and calmness). We hypothesized that the associations of speech features with negative affect are similar to those for depression severity and that the associations of speech features with the other momentary affective states listed follow the opposite pattern.

## Methods

### Sample

We used a data set that was collected as part of a pilot study (Sleep Deprivation and Gene Expression [SLEDGE II]; German Clinical Trials Register: DRKS00022025) gathering digital phenotypes and multiomics data in a clinical sample undergoing SDT at the Central Institute of Mental Health in Mannheim, Germany. A total of 30 inpatients experiencing acute depressive episodes were enrolled in the study. The patients were diagnosed according to the *International Classification of Diseases, Tenth Revision* (ICD-10), codes by the senior clinician at admittance to the hospital. All patients received treatment as usual, which also included SDT (for a list of medications, refer to Textbox S1 in Multimedia Appendix 1). Exclusion criteria were comorbid substance use disorders or personality disorders. From this sample of 30 patients, the complete data sets of 8 (27%) patients were excluded from the final analyses (n=4, 50% did not record any videos; n=1, 13% did not say anything during the videos [23 videos]; n=2, 25% had no sound recorded in the videos owing to technical issues [30 recordings]; and n=1, 13% recorded only 2 videos); thus, the final sample consisted of 22 (73%) patients (n=12, 55% male) aged between 18 and 63 (mean 33.5, SD 12.4; median 29, IQR 23.25-42.75) years.

### Ethical Considerations

The study was approved by the Ethics Committee II of the Medical Faculty Mannheim, University of Heidelberg (2013-563N-MA). All patients received detailed information about the aims and procedures of the study and provided informed consent. Patients could withdraw from the study at any time and did not receive any compensation for participation. Data was deidentified to ensure privacy.

### Study Procedure

Patients were given a study smartphone (Nokia 4.2 or Samsung Galaxy J7) at the beginning of the study (day 0), instructed on how to use it, and (if necessary) performed test runs supervised by the study personnel. A telephone number for technical support and an information sheet regarding the ambulatory assessment procedure were handed out. Data were collected using movisensXS software (movisens GmbH) [38]. Patients underwent SDT as part of their depression treatment, which involves staying awake for approximately 36 hours. Treatment effect and relapse can be measured in a matter of 4 days [37], thus ensuring a maximum of within-person variance in the data set. After at least 1 day of baseline assessment (day 0), SDT was conducted on day 1. Patients stayed awake from 6 AM on day 1 to 6 PM on day 2. Recovery sleep was allowed from 6 PM on day 2 until 1 AM on day 3. Data were collected before, during, and after SDT for up to 26 days. In the first week of the study, smartphones sent prompts 3 times per day (morning, afternoon, and evening); in addition, self-initiated assessments were possible to report specific events or to catch up with missed assessments. To reduce the burden on patients, the sampling schema was altered to 2 prompts per day (morning and evening). With each prompt, patients were requested to fill out items concerning their affective state and to record a selfie video reporting how they felt currently. Patients returned the smartphone at the end of the study. The study personnel uploaded the data from the smartphones to the movisensXS platform [38] and then downloaded the data for analysis.

### Ambulatory Assessment: eDiary Ratings and Selfie Videos

The data set contains 3 sets of momentary assessments in the form of eDiary ratings at each prompt (Textboxes S2-S4 in Multimedia Appendix 1): (1) the short version of the Allgemeine Depressionsskala (ADS-K) [39] adapted to momentary assessment with 14 items on depressive mood rated on a scale ranging from 0=*rarely* to 3=*mostly* (we left out the item regarding sleep from the original questionnaire because its inclusion was not reasonable in the momentary assessment design); (2) a total of 15 positive (cheerful, content, energetic, enthusiastic, relaxed, and happy) and negative (lonely, sad, insecure, anxious, depressed, low-spirited, guilty, distrustful, and irritable) affect items [40] rated on a 5-point Likert scale ranging from 1=*not at all* to 5=*very much*; and (3) a 6-item short version of the Multidimensional Mood Questionnaire (MDMQ) [41] capturing time-varying momentary fluctuations in daily life on the affect dimensions of valence (*unwell* to *well* and *discontent* to *content*), energetic arousal (*without energy* to *full of energy* and *tired* to *awake*), and calmness (*tense* to *relaxed* and *agitated* to *calm*). The items were presented on visual analog scales with 2 poles and a slider from 0 to 100. For each of the constructs, we computed mean values per scale, resulting in 6 outcome variables (depressive symptoms, positive affect, negative affect, valence, energetic arousal, and calmness). For the ADS-K, we also report sum scores as described in the tool’s manual; however, to increase comparability among outcomes, we used the mean value for analyses. If necessary, we recoded items such that higher values indicated a (1) higher intensity of depressive symptoms, (2) higher positive affect, (3) higher negative affect, (4) higher positive valence, (5) higher energetic arousal, and (6) higher calmness.

In addition to the aforementioned eDiary ratings, patients were requested to record selfie videos with the following instructions: “Please keep the camera stable during the recording and record your whole face. Please describe in 10-20 seconds how you currently feel.”

### Clinical Assessments

The Montgomery–Åsberg Depression Rating Scale (MADRS) [42] was completed in the morning at 4 time points (baseline, morning before sleep deprivation, 1 week after sleep deprivation, and 2 weeks after sleep deprivation) and once at midday (the day after sleep deprivation night). The MADRS is a 10-item expert assessment of depressive symptom severity over the past week, with items rated on a 7-point scale ranging from 0 to 6; higher scores indicate higher severity.

### Data Preprocessing

The data set contained 899 selfie videos in mp4 format. The full set of videos of 4 (13%) of the 30 patients had to be excluded owing to the reasons mentioned previously (55/899, 6.1%) and additional 2 videos had to be excluded because of technical damage (2/899, 0.02%). As our research questions focused on audio data (not visual data), we extracted the audio tracks of the remaining 842 (93.66%) from the original 899 selfie videos using the *ffmpeg* package in Python and archived them as wav files (sampling rate: 48 kHz; mono=1 channel). We excluded test runs (14/842, 1.7%), accidental short recordings with no content (29/842, 3.4%), recordings during which the microphone was masked by the patient (27/842, 3.2%), and assessments in which 1 of the 2 corresponding assessments (speech or affective state) was missing (18/842, 2.1%). In addition, if 2 consecutive assessments were <15 minutes apart from each other, only the first assessment was kept unless its audio quality was insufficient or only the second assessment included assessments of affective states; in such cases, the second assessment was kept (21/842, 2.5%). We also excluded recordings with background noise that restricted speech intelligibility (9/842, 1.1%) or that included the speech of third parties (8/842, 1%). We filtered the remaining recordings (716/842, 85%) using DeepFilterNet2 [43] to remove background noise.

### Acoustic Features

For our main analyses, we focused on the acoustic features *pitch variability*, *speech pauses*, and *speech rate* (Table 1). We restricted the number of features to limit α error inflation and selected specifically these 3 features because they revealed sufficient empirical support to warrant an explicit hypothesis. We extracted acoustic features of the final recordings (n=716) using the functionals (v02) of the extended Geneva Minimalistic Acoustic Parameter Set (eGeMAPS) [44] of the open-source toolkit openSMILE implemented in Python [45,46]. eGeMAPS is a minimalistic set of acoustic features recommended for clinical speech analysis; it helps to guarantee comparability between studies, given the proliferation of speech features. Features related to frequency, energy, spectrum, and tempo are included in the set. *Pitch variability* is represented by the SD of the logarithmic fundamental frequency (F0) on a semitone frequency scale starting at 27.5 Hz and measured in hertz. F0 is the lowest frequency of the speech signal and is perceived as *pitch*. *Speech pauses* are approximated as the mean length of unvoiced regions (F0=0) measured in seconds. With respect to *speech rate*, a transcription of the recordings is necessary, which we obtained using an automatic speech recognition system according to published procedures [47]. We corrected the transcripts manually. To determine *speech rate,* we calculated the ratio of words divided by the duration of the voice sample.

**Table 1:** Overview of extracted speech features.

Speech feature	Technical feature	Explanation
Pitch variability	F0semitoneFrom27.5Hz_sma3nz_stddevNorm	SD of the F0 perceived as the extent to which a person’s *pitch* changes (in Hz)
Speech pauses	MeanUnvoicedSegmentLength	Mean of the length of unvoiced regions approximating silent parts of the speech sample (in seconds)
Speech rate	Words per second	Ratio of words counted on the basis of the automatically transcribed and manually corrected text divided by the duration of the speech sample

Beside our main analyses based on *pitch variability*, *speech pauses*, and *speech rate*, we decided to integrate further eGeMAPS features in an exploratory analysis. These features have been recommended in the context of affective states in particular because they contain additional cepstral and dynamic features [44]. We included the following features in the exploratory analyses: for voiced and unvoiced regions together, the mean and SD of the mel-frequency cepstral coefficients (MFCCs) 1 to 4 and spectral flux difference of the spectra of 2 consecutive frames; for voiced regions, the formant 2 to 3 bandwidths along with spectral flux and MFCCs 1 to 4; and for unvoiced regions, the mean and SD of the spectral flux [44].

### Statistical Analysis

In addition to the *mean*, *SD*, and *range,* we present *min* and *max* as the mean of all patients’ minimum and maximum scores, respectively, of each parameter throughout the whole study. Moreover, following the recommendations by Snijders and Bosker [48], we computed Pearson correlation analyses with person-mean–centered variables to evaluate the relationship between affective scores and speech features. To generate person-mean–centered variables, we subtracted the individual’s mean from their score, which represents the variation around the individual’s mean.

To evaluate psychometric properties, we calculated McDonald ω as the reliability coefficient using the *multilevelTools* package in R. For the MDMQ subscales, we used the *misty* package in R to calculate the Spearman-Brown corrected correlation coefficients because the subscales consist of only 2 items [49]. For the MADRS score at the time of inclusion, we calculated Cronbach α using the *psych* package in R.

To analyze the within-person association of speech features and subjectively evaluated affective states, we used multilevel modeling [48] using the *nlme* package in R. Multilevel modeling offers two specific advantages for the given data: (1) separation of within-person effects from between-person effects and (2) allowing and considering different numbers of assessments per patient. Before the analyses, we centered time-variant level-1 predictors (*pitch variability*, *speech pauses*, and *speech rate*) at the person level and included the predictors *time* and *time²* in minutes (each centered at 2 PM) as covariates. To facilitate the comparison of the magnitude of effects among different predictors, we report standardized beta coefficients (standardized β) according to the recommendations by Hox and van de Schoot [50] following the equation: standardized β = β × (SD_predictor_ / SD_outcome_). We further calculated Hox *R*² values according to the recommendation by Hox and Maas [51] following the equation: *R*^²^_Hox_ = (σ^²^_null_ − σ^²^_model_) / σ^²^_null_. We set the α level at 5% and applied Bonferroni corrections for exploratory analyses (α_adj_=.002). We performed all analyses in R (version 4.2.1, 2022-06-23).

Our analyses can be split into 4 parts: the calculation of intraclass correlation coefficients (ICCs); separate models with all speech features as predictors and all affective scores as outcomes; combined models with all speech features as simultaneous predictors; and exploratory analyses, including additional speech features. Specifically, we first descriptively investigated whether our study procedure resulted in sufficient within-person variance. For this purpose, we calculated ICCs, including all momentary affective ratings and speech recordings, regardless of whether they were assessed before, during, or after SDT. In general, the ICC indicates the amount of between-person variance in unconditional (null) models. The 2-level models analyzed contained repeated measures (level 1) that were nested within patients (level 2). The second step contained our main analysis: we calculated separate models for each speech feature (*pitch variability* [model set 1], *speech pauses* [model set 2], and *speech rate* [model set 3]) and each affective state (depression severity [ADS-K], positive affect, negative affect, valence, energetic arousal, and calmness), resulting in 18 models. In the third step, to evaluate the relative significance of *pitch variability*, *speech pauses*, and *speech rate*, we constructed combined models for each of the affective scores, including all 3 features simultaneously (6 models). In the fourth step, exploratory analyses were conducted with the inclusion of 24 additional speech features from eGeMAPS (Textbox S5 in Multimedia Appendix 1). These features were used as predictors for each of the affective scores separately.

## Results

### Descriptive Statistics

We included 716 speech-state pairs (mean 32, SD 19.83 per patient) in the final analysis. The mean MADRS score at the time of inclusion assessment was 30.1 (SD 5.8). This corresponds to 18 (82%) patients with moderate depression and 4 patients (18%) with severe depression out of 22 patients at study inclusion.

Regarding depressive symptoms (ADS-K; scale 0-3), patients had a mean score of 1.2 (SD 0.6; min 0.7, max 2.0) and a mean sum score of 16.9 (SD 8.1; min 9.6, max 26.1). At inclusion, the mean ADS-K score was 1.4 (SD 0.6; range 0.4-2.8), and the mean sum score was 20.0 (SD 8.4; range 6-39). For positive and negative affect (scale 1-5), the mean scores were 2.1 (SD 0.8; min 1.3, max 3.1) and 2.3 (SD 1.0; min 1.4, max 3.9), respectively; on the MDMQ (scale 1-100) valence subscale, the mean score was 44.9 (SD 21.5; min 9.4, max 67.5); on the energetic arousal subscale, the mean score was 41.7 (SD 21.0; min 16.4, max 62.7); and on the calmness subscale, the mean score was 43.8 (SD 22.8; min 6.9, max 70.7). The ICCs were 0.47 for the ADS-K, 0.45 for positive affect, 0.59 for negative affect, 0.27 for energetic arousal, 0.25 for valence and 0.40 for calmness, that is, the following amount of variance in the momentary assessments can be attributed to within-person fluctuations: 53% for the ADS-K, 55% for positive affect, 41% for negative affect, 73% for energetic arousal, 75% for valence, and 60% for calmness.

Regarding speech features, the mean *pitch variability* was 0.32 Hz (SD 0.09; min 0.14, max 0.44), the mean *speech pause* length was 0.26 seconds (SD 0.12; min 0.17, max 0.47), and the mean *speech rate* was 1.77 words per second (SD 0.57; min 1.16, max 2.75). The ICCs were 0.66 for *pitch variability,* 0.36 for *speech pauses*, and 0.57 for *speech rate*. This corresponds to the following amount of variance in the speech feature assessments that can be attributed to within-person fluctuations: 34% for *pitch variability*, 64% for *speech pauses*, and 43% for *speech rate*.

Correlational analyses (Figure S1 in Multimedia Appendix 1) included between 698 and 716 observations depending upon the specific pairing. We found correlations among and between affective scores and speech features, except for *pitch variability* and *speech rate*, neither of which correlated with negative affect and calmness; in addition, there was no correlation between *pitch variability* and *speech rate*. Specifically, ADS-K scores correlated negatively with positive affect, all MDMQ subscales, and *speech rate* and correlated positively with negative affect, *pitch variability*, and *speech pauses*. Negative affect showed the same pattern, except for the pairings with *pitch variability* and *speech rate*, for which no correlations were found. Regarding positive affect, we found the opposite correlation pattern, that is, positive correlations with all MDMQ subscales and *speech rate* and negative correlations with *pitch variability* and *speech pauses*. The MDMQ subscales showed the same relationships as positive affect, except for the pairing between calmness and *pitch variability* and *speech rate*, for which no correlations were found. Within speech features, we found a negative correlation between *pitch variability* and *speech pauses*, no correlation between *pitch variability* and *speech rate*, and a negative correlation between *speech pauses* and *speech rate.* Overall, correlations among affective scores were strong (*r*>0.5). Correlations among speech features as well as between affective scores and speech features were weak (*r*<0.2), except for a strong negative correlation between *speech pauses* and *speech rate.*

The psychometric properties for momentary affective ratings were good to excellent. Specifically, McDonald ω values [52] were 0.87 (within-person) and 0.90 (between-person) for depressive symptoms (ADS-K), 0.87 (within-person) and 0.95 (between-person) for positive affect, and 0.87 (within-person) and 0.96 (between-person) for negative affect. The Spearman-Brown coefficients were 0.83 (within-person) and 0.94 (between-person) for valence, 0.74 (within-person) and 0.89 (between-person) for energetic arousal, and 0.74 (within-person) and 0.89 (between-person) for calmness. Cronbach α for the MADRS score at the time of inclusion was acceptable (.67).

### Association Between Speech Features and Momentary Affective Scores

#### Overview

In Tables 2 and 3, we present the fixed effects of *pitch variability*, *speech pauses*, and *speech rate* separately for each affective state. Details, including the effects of time and time², are presented in Table S1 in Multimedia Appendix 1.

**Table 2:** Multilevel linear regression analysis to predict depression and positive and negative affect: fixed effects of pitch variability, speech pauses, and speech rate.

Predictors			Outcome																												
			ADS-K^a^										Positive affect										Negative affect								
			β		Standardized β		SE		*R*²_Hox_, %	*P* value		β		Standardized β		SE		*R*²_Hox_, %		*P* value		β			Standardized β		SE		*R*²_Hox_, %		*P* value
**Model set 1**																															
	Intercept	1.27		N/A^b^		0.10		N/A		<.001	2.10			N/A	0.13		N/A		<.001		2.45			N/A		0.16		N/A		<.001	
	Pitch variability	.88		.14		0.32		1		.007	−1.50			−.18	0.42		1		<.001		.85			.08		0.43		1		.05	
**Model set 2**																															
	Intercept	1.27		N/A		0.10		N/A		<.001	2.09			N/A	0.13		N/A		<.001		2.46			N/A		0.16		N/A		<.001	
	Speech pauses	.52		.10		0.18		1		.005	−1.16			−.17	0.24		17		<.001		.76			.09		0.25		2		.002	
**Model set 3**																															
	Intercept	1.27		N/A		0.10		N/A		<.001	2.10			N/A	0.13		N/A		<.001		2.45			N/A		0.16		N/A		<.001	
	Speech rate	−.11		−.10		0.05		<1		.02	.26			.18	0.06		2		<.001		−.13			−.08		0.07		1		.04	

^a^ADS-K: Allgemeine Depressionsskala.

^b^N/A: not applicable.

**Table 3:** Multilevel linear regression analysis to predict valence, energetic arousal, and calmness: fixed effects of pitch variability, speech pauses, and speech rate.

Predictors		Outcome																
		Valence						Energetic arousal						Calmness				
		β	Standardized β	SE	*R*²_Hox_, %	*P* value	β		Standardized β	SE	*R*²_Hox_, %	*P* value	β		Standardized β	SE	*R*²_Hox_, %	*P* value
**Model set 1**																		
	Intercept	43.72	N/A^a^	2.70	N/A	<.001	42.82		N/A	2.71	N/A	<.001	40.97		N/A	3.39	N/A	<.001
	Pitch variability	−36.50	−.16	13.61	1	.008	−33.21		−.15	12.48	1	<.001	−11.52		−.05	12.82	<1	.37
**Model set 2**																		
	Intercept	43.26	N/A	2.69	N/A	<.001	42.71		N/A	2.71	N/A	<.001	40.58		N/A	3.39	N/A	<.001
	Speech pauses	−34.06	−.19	7.71	3	<.001	−14.06		−.08	7.14	1	.049	−24.27		−.12	7.27	5	<.001
**Model set 3**																		
	Intercept	43.56	N/A	2.70	N/A	<.001	42.77		N/A	2.71	N/A	<.001	40.86		N/A	3.39	N/A	<.001
	Speech rate	6.49	.17	2.03	2	.001	4.13		.11	1.87	1	.03	3.43		.09	1.91	5	.07

^a^N/A: not applicable.

#### ADS-K Scores

In the column entitled *ADS-K* (Table 2), we report the results of all models with ADS-K scores as the outcome. *Pitch variability* (standardized β=.14; *P*=.007), *speech pauses* (standardized β=.10; *P*=.005), and *speech rate* (standardized β=−.10; *P*=.02) were significantly associated with the ADS-K score, indicating that higher *pitch variability*, longer *speech pauses*, and lower *speech rate* are associated with more severe depressive symptomatology.

#### Positive and Negative Affect

In the columns entitled *Positive affect* and *Negative affect* (Table 2)*,* we show results for positive affect and negative affect, respectively, as outcomes. *Pitch variability* (standardized β=−.18; *P*<.001), *speech pauses* (standardized β=−.17; *P*<.001), and *speech rate* (standardized β=.18; *P*<.001) were significantly associated with positive affect, indicating that lower *pitch variability*, shorter *speech pauses*, and higher *speech rate* are associated with higher positive affect. The associations between negative affect and speech features were in the opposite direction of the associations between positive affect and the speech features just presented: *speech pauses* (standardized β=.09; *P*=.002) and *speech rate* (standardized β=−.08; *P*=.04) were significantly associated with negative affect, indicating that longer *speech pauses* and lower *speech rate* are associated with higher negative affect. We further found a trend with respect to the association between *pitch variability* and negative affect, but this result was not statistically significant (standardized β=.08; *P*=.05). In addition, we found trends with respect to the associations between negative affect and time and negative affect and time², specifically in the models that included *pitch variability* (time: standardized β=.04; *P*=.08), *speech pauses* (time: standardized β=.04; *P*=.08; time²: standardized β<.01; *P*=.06), and *speech rate* (time: standardized β=.04; *P*=.09), but these results were not statistically significant.

#### MDMQ Results

In the columns entitled *Valence*, *Energetic arousal*, and *Calmness* (Table 3), we present the results for the MDMQ. *Pitch variability* (standardized β=−.16; *P*=.008), *speech pauses* (standardized β=−.19; *P*<.001), and *speech rate* (standardized β=.17; *P*=.001) were significantly associated with valence, indicating that lower *pitch variability*, shorter *speech pauses*, and higher *speech rate* are associated with higher (ie, positive) valence. In the model that included valence and *speech pauses,* we found a significant association between time² and valence (standardized β<.001; *P*=.03)*.* In addition, we found trends with respect to the associations between valence and time², specifically in the models that included *pitch variability* (time: standardized β<.01; *P*=.098) and *speech rate* (time: standardized β<.01; *P*=.07), but these results were not statistically significant. Moreover, *pitch variability* (standardized β=−.15; *P*<.001), *speech pauses* (standardized β=−.08; *P*=.049), and *speech rate* (standardized β=.11; *P*=.03) were significantly associated with energetic arousal, indicating that lower *pitch variability*, shorter *speech pauses*, and higher *speech rate* are associated with higher energetic arousal. In all model combinations of energetic arousal and each speech feature, we found significant associations between time and energetic arousal (standardized β=−.11; *P*<.001) and time² and energetic arousal (standardized β<.01; *P*<.001). Furthermore, *speech pauses* (standardized β=−.12; *P*<.001) were significantly associated with calmness, indicating that shorter *speech pauses* are associated with greater calmness. In all model combinations of calmness and each speech feature, we found significant associations between time² and calmness (standardized β<.01; *P*= .013 for *pitch variability*, *P*=.003 for *speech pauses*; *P*=.009 for *speech rate*). In addition, we found a trend with respect to the association between *speech rate* and calmness (standardized β=.09; *P*=.07), but this result was not statistically significant.

### Combined Models

In Tables 4 and 5, we display the results for the combined models that included all 3 speech features. In the model of ADS-K scores, associations with *pitch variability* (standardized β=.17; *P*<.001) and *speech pauses* (standardized β=.12; *P*=.01) remained statistically significant. Regarding positive affect, associations with *pitch variability* (standardized β=−.23; *P*<.001) and *speech pauses* (standardized β=−.19; *P*<.001) remained statistically significant. We further found a trend regarding the association between positive affect and time (standardized β=−.05; *P*=.09), but this result was not statistically significant. Regarding negative affect, associations with *pitch variability* (standardized β=.12; *P*=.008), *speech pauses* (standardized β=.12; *P*=.005), time (standardized β=.05; *P*=.03), and time² (standardized β<.01; *P*=.03) remained statistically significant. In the model of valence, associations with *pitch variability* (standardized β=−.22; *P*<.001), *speech pauses* (standardized β=.22; *P*<.001), and time² (standardized β<.01; *P*=.01) remained statistically significant. Regarding energetic arousal, associations with *pitch variability* (standardized β=−.17; *P*=.003), time (standardized β=.12; *P*<.001), and time² (standardized β<.01; *P*<.001) remained statistically significant. Regarding calmness, associations with *speech pauses* (standardized β=−.17; *P*=.002) and time² (standardized β<.01; *P*=.002) remained statistically significant. We further found a trend for the association between calmness and *pitch variability* (standardized β=.09; *P*=.097), but this result was not statistically significant.

**Table 4:** Multilevel linear regression analysis to predict momentary depression, positive affect, and negative affect: fixed effects of the combined models that included pitch variability, speech pauses, speech rate, time, and time².

Predictors^a^	Outcome													
	ADS-K^b^					Positive affect					Negative affect			
	β	Standardized β	SE	*P* value	β		Standardized β	SE	*P* value	β		Standardized β	SE	*P* value
Intercept	1.28	N/A^c^	0.10	<.001	2.08		N/A	0.13	<.001	2.47		N/A	0.16	<.001
Time	<.01	.02	<0.01	.42	<−.01		−.05	<0.01	.09	<.01		.05	<0.01	.03
Time²	<.01	<.001	<0.01	.44	<.01		<.001	<0.01	.31	<.01		<.01	<0.01	.03
Pitch variability	1.11	.17	0.33	<.001	−1.96		−.23	0.43	<.001	1.19		.12	0.45	.008
Speech pauses	.64	.12	0.26	.01	−1.29		−.19	0.33	<.001	.99		.12	0.35	.005
Speech rate	<−.01	<.001	0.07	.99	.04		.03	0.09	.66	.04		.02	0.09	.68

^a^*R*²_Hox_ for ADS-K=2%, for positive affect=6%, and for negative affect=2%.

^b^ADS-K: Allgemeine Depressionsskala.

^c^N/A: not applicable.

**Table 5:** Multilevel linear regression analysis to predict momentary valence, energetic arousal, and calmness: fixed effects of the combined models that included pitch variability, speech pauses, speech rate, time, and time².

Predictors^a^	Outcome												
	Valence				Energetic arousal					Calmness			
	β	Standardized β	SE	*P* value	β	Standardized β	SE	*P* value	β		Standardized β	SE	*P* value
Intercept	42.95	N/A^b^	2.68	<.001	42.48	N/A	2.71	<.001	40.45		N/A	3.38	<.001
Time	<.01	.03	<0.01	.48	<−.01	.12	<0.01	<.001	<−.01		.01	<0.01	.89
Time²	<.01	<.01	<0.01	.01	<.01	<.01	<0.01	<.001	<.01		<.01	<0.01	.002
Pitch variability	−49.01	−.22	13.76	<.001	−37.74	−.17	12.78	.003	−21.75		.09	13.07	.097
Speech pauses	−41.01	.22	10.73	<.001	−12.97	.07	9.97	.19	−32.53		−.17	10.20	.002
Speech rate	−.64	.02	2.76	.82	1.96	.05	2.56	.44	−2.28		.06	2.62	.38

^a^*R*²_Hox_ for valence=4%, for energetic arousal=5%, and for calmness=2%.

^b^N/A: not applicable.

### Exploratory Analysis

Analyzing additional speech features, we found significant associations of the *equivalent sound level*, the mean of *spectral flux*, and the mean of *spectral flux of voiced regions only*, individually, with all affective scores (Table S2 in Multimedia Appendix 1). With respect to *equivalent sound level,* this indicates that louder voice samples were linked to improved affective states (ADS-K: standardized β=−.30; positive affect: standardized β=.34; negative affect: standardized β=−.21; valence: standardized β=.29; energetic arousal: standardized β=.26; and calmness: standardized β=.19); with respect to the mean of *spectral flux*, this indicates that a faster change in the spectrum was linked to better affective states (ADS-K: standardized β=−.22, positive affect: standardized β=.28, negative affect: standardized β=−.15, valence: standardized β=.21, energetic arousal: standardized β=.17, and calmness: standardized β=.27); and with respect to the mean of *spectral flux of voiced regions only,* this indicates that a faster change in the spectrum in voiced regions was linked to better affective states (ADS-K: standardized β=−.23, positive affect: standardized β=.28, negative affect: standardized β=−.15, valence: standardized β=.20, energetic arousal: standardized β=.20, and calmness: standardized β=.16). Regarding the additional speech features, the following significant associations were found: the mean of *spectral flux of unvoiced regions only* was associated with positive affect, indicating that a faster change in the spectrum in unvoiced regions was linked to improved positive affect (standardized β=.13); and the mean of the *MFCC 2 of voiced regions only* was significantly associated with energetic arousal, indicating that a higher mean was linked to lower energetic arousal (standardized β=−.15). Furthermore, we revealed a significant association between the SD of the *MFCC 4 of voiced regions only* ADS-K scores (standardized β=.13) as well as positive affect (standardized β=−.10) and negative affect (standardized β=.09). Specifically, smaller SDs were linked to higher positive affect, reduced negative affect, and lower ADS-K scores.

## Discussion

### Principal Findings

This is the first study to investigate whether speech features are associated with depression severity and momentary affective states in a longitudinal data set of patients with a depressive episode undergoing SDT. Our findings showed that lower *pitch variability*, higher *speech rate*, and shorter *speech pauses* were associated with better momentary states (ie, lower depression severity; higher positive affect and lower negative affect; and higher positive valence, energetic arousal, and calmness), supporting prior clinical observations with innovative methods applied to an intensive longitudinal data set.

Lower depression severity was accompanied by shorter *speech pauses*. This is in line with past research findings reporting that shorter *speech pauses* were associated with lower depression severity [31-33,36]. Our findings extend prior results because we also found an association between *speech pauses* and affective states more broadly, not limited to depressed mood. Regarding *speech rate*, we revealed associations with depression severity and all other affective state scales except for calmness. In particular, we found that higher *speech rate* was associated with lower depression symptomatology and lower negative affect, higher positive affect, higher positive valence, and higher energetic arousal. This is in line with prior research [31-33,35], in which a higher *speech rate* was found for patients who benefited from treatment.

Regarding *pitch variability*, we found support for our hypothesis that *pitch variability* changes with depression severity; more precisely, lower *pitch variability* was associated with lower depression symptomatology. This is in line with the studies by Quatieri and Malyska [34] and Horwitz et al [35], where a positive correlation between *pitch variability* and depression severity was found. However, the results reported in the studies by Mundt et al [31] and Yang et al [36] contrasted with ours and those found in the studies by Quatieri and Malyska [34] and Horwitz et al [35], that is, that higher *pitch variability* was associated with lower depression severity. A possible explanation for contradictory results in major depression are the heterogeneity of (1) the depression phenotype per se because diagnosis criteria include >400 possible symptom combinations [53,54]; and (2) the questionnaires, assessment approaches, statistical analyses, and speech feature extraction tools used in these studies. The within-person research design approach underlying our data set addressed the heterogeneity of the depression phenotype at least partially. Furthermore, we analyzed free speech collected naturally in a selfie task, whereas in the study by Mundt et al [31], read speech was used in the analyses. In line with what is suggested in the study by Quatieri and Malyska [34], this could also be a reason for the contradictory results. However, because assessing within-person fluctuations in daily life increases ecological validity, we regard our results as an important contribution.

Observing the full picture of associations, we note that the results for all 3 speech features are similar and do not provide evidence of specific associations (eg, association of 1 specific speech feature with 1 specific momentary affective state), showing no distinct patterns of momentary states for each speech feature. This is reasonable because the constructs overlap in content (eg, patients experiencing depression experience higher negative affect and lower positive affect).

In terms of the combined models evaluating the relative importance of the features, we found that in the 4 models (ADS-K, valence, positive affect, and negative affect) both *pitch variability* and *speech pauses* remained significant, whereas *speech rate* did not. *Pitch variability* remained the only significant parameter in the model of energetic arousal, and *speech pauses* remained the only significant parameter in the model of calmness. This suggests that *pitch variability* and *speech pauses* are speech features rather independent of each other, whereas the high correlation between *speech pauses* and *speech rate* might account for the fact that only 1 of these features (in this case, *speech pauses)* remained a significant predictor.

### Limitations

First, this study examined a limited set of 3 speech features. Instead of applying brute force methods involving thousands of technical speech features, we selected speech features based on previous work and with high face validity, restricting the scope of our analysis. Although we did expand our scope of features in the exploratory analysis, it is very likely that other configurations and features (eg, the *ComParE feature set* containing 6373 features [55]) might also be predictive of affective states. Future work is needed to compare theory-driven approaches with brute force data-driven machine learning methods to find the best possible combination of speech features also considering aspects of computational power. However, selecting the features on a theoretical basis and restricting their pure number limits alpha error inflation and should increase replicability.

Second, although the sample size of our study was limited, this was a true within-person design with many data points per patient. In addition, we regard this study as a pilot study providing important indications regarding feasibility in a clinical context. As some patients dropped out of the study, and some recordings had to be excluded, in future studies, data collection needs to be integrated better into clinical routines. Moreover, the instructions for patients may need to be revised to reduce the likelihood of missing data and recording errors. However, the data set at hand is still unique in the relatively high number of assessments per patient and the applied SDT, which yielded meaningful variation in the depression severity within a short time period. From a theoretical perspective, it is crucial to emphasize that to uncover existing relations among variables, meaningful variance in both parameters is needed.

Third and last, selfie videos were recorded in a clinical environment, which may limit generalizability to other contexts. In future studies, ambulant patients could be integrated and other environments explored to evaluate the replicability of the results. However, our approach, which involved sampling free speech, offers higher ecological validity to reading standardized text paragraphs because it provides a closer representation of people’s everyday lives. The development of passive sensing will be helpful in this context (ie, the random assessment of audio bits in an ecological environment). To date, automated passive voice recordings in nonprotected environments have been restricted in 2-party consent states, such as Germany. However, in single-party consent states, a few speech-related applications can be used *in the wild* (eg, the Electronically Activated Recorder [56]). Although the development of technical devices is ongoing, future studies will have to consider ethical issues related to voice recording in natural settings (eg, ensuring that no third parties who did not give informed consent are recorded).

### Conclusions

Our study provides evidence that fluctuations in the speech features *pitch variability*, *speech pauses*, and *speech rate* are associated with fluctuations in depression severity and other momentary affect states. Notably, the data were collected from clinically diagnosed patients (no subclinical sample or staged emotions) experiencing an acute depressive episode. A particularly important advantage is that our longitudinal ambulatory assessment data set ensured a maximum of within-person dynamics of depressive parameters within a short time period by applying a sleep deprivation intervention design. This is of great importance because future technology will try to predict upcoming depressive episodes on an individual level and will need information on within-person trajectories. For the development of such tailored precision medicine tools, *pitch variability*, *speech pauses*, and *speech rate* present promising features. Our research is a step forward on the path to developing an automated depression monitoring system, facilitating individually tailored treatments and increased patient empowerment.

## Appendix

Multimedia Appendix 1 Supplementary material.

## Abbreviations

ADS-K: Allgemeine Depressionsskala
eGeMAPS: extended Geneva Minimalistic Acoustic Parameter Set
F0: fundamental frequency
ICC: intraclass correlation coefficient
ICD-10: International Classification of Diseases, Tenth Revision
MADRS: Montgomery–Åsberg Depression Rating Scale
MDMQ: Multidimensional Mood Questionnaire
MFCC: mel-frequency cepstral coefficient
openSMILE: open-source Speech and Music Interpretation by Large-Space Extraction
SDT: sleep deprivation therapy
SLEDGE II: Sleep Deprivation and Gene Expression
