# Supplementary material for: Speech Features as Predictors of Momentary Depression Severity in Patients With Depressive Disorder Undergoing Sleep Deprivation Therapy: Ambulatory Assessment Pilot Study
Source: JMIR Ment Health. 2024 Jan 18;11:e49222. doi: 10.2196/49222 (PMC10835582; doi:10.2196/49222)
Supplement: Multimedia Appendix 1 [file mental_v11i1e49222_app1.pdf]

Multimedia Appendix

Textbox S1. List of medications.

Patients received guideline-compliant pharmacotherapy for depression. Five participants received monotherapy with an antidepressant agent (n=1 sertraline (SSRI); n=2 venlafaxine (SSNRI); n=1 bupropion and n=1 trazodone); thirteen patients were treated with two antidepressants (n=4 venlafaxine and trazodone; n=4 bupropion and trazodone; n=2 venlafaxine and mirtazapine; n=1 bupropione and mirtazapine; n=1 sertraline and trazodone; n=1 venlafaxine and amitriptyline; two patients were prescribed an antidepressant plus augmentation therapy (n=1 bupropion and quetiapine; n=1 venlafaxine and pregablin); one patient received a quindruple combination of two antidepressants medication (bupropion and venlafaxine) and augmentation with quetiapine, lamotrigine and pregabalin. Above-mentioned sedative, respectively, sleep-inducing medication (trazodone, amitriptyline, quetiapine, pregabalin,) was paused before sleep depreviation night.

Sertraline = SSRI

Venlafaxine = SSNRI

Mirtazapine = NaSSA

Bupropion = SNDRI

Agomelatine = MASSA

Amitriptyline = TCA

Trazodone = chemically different antidepressant

Wadle LM, Ebner-Priemer UW, Foo JC, et al. Speech Features as Predictors of Momentary Depression Severity in Patients With Depressive Disorder Undergoing Sleep Deprivation Therapy: Ambulatory Assessment Pilot Study. JMIR Mental Health. 2024;11:e49222.
doi:10.2196/49222

Textbox S2. ADS-K items with englisch translations in italics

1) Während der letzten Minuten haben mich Dinge beunruhigt, die mir sonst nichts ausmachen.
During the last few minutes, things that normally don't bother me worried me.

2) Während der letzten Minuten konnte ich meine trübsinnige Laune nicht loswerden, obwohl mich meine Freunde / Familie / Mitpatienten versuchten aufzumuntern.
During the last few minutes, I couldn't get rid of my gloomy mood, although my friends / family / fellow patients tried to cheer me up.

3) Während der letzten Minuten hatte ich Mühe mich zu konzentrieren.
During the last few minutes I had trouble concentrating.

4) Während der letzten Minuten war ich deprimiert / niedergeschlagen.
During the last few minutes I was depressed / down.

5) Während der letzten Minuten war alles anstrengend für mich.
During the last minutes everything was exhausting for me.

6) Während der letzten Minuten dachte ich, mein Leben ist ein einziger Fehlschlag.
During the last minutes I thought my life was one big failure.

7) Während der letzten Minuten hatte ich Angst.
During the last minutes I was afraid.

8) Während der letzten Minuten war ich fröhlich gestimmt.
During the last minutes I was in a cheerful mood.

9) Während der letzten Minuten habe ich weniger als sonst geredet.
During the last minutes I talked less than usual.

10) Während der letzten Minuten fühlte ich mich einsam.
During the last minutes I felt lonely.

11) Während der letzten Minuten habe ich das Leben genossen.
During the last minutes I enjoyed life.

12) Während der letzten Minuten war ich traurig.
During the last minutes I felt sad.

13) Während der letzten Minuten hatte ich das Gefühl, dass mich die Leute nicht leiden können.
During the last minutes I felt that people didn't like me.

14) Während der letzten Minuten konnte ich mich zu nichts aufraffen.
During the last minutes I couldn't get myself up to do anything.

Textbox S3. MDMQ items with english translations in italics.

Im Moment fühle ich mich …
At the moment I feel ...

unzufrieden – zufrieden
discontent- content

unwohl – wohl
unwell - well

müde – wach
tired - awake

Im Moment fühle ich mich …
At the moment I feel ...

energielos – energiegeladen
without energy - full of energy

unruhig – ruhig
agitated - calm

angespannt – entspannt
tense - relaxed

Textbox S4. Positive and negative affect items with english translations in italics

Im Moment fühle ich mich
fröhlich / zufrieden / tatkräftig / enthusiastisch / entspannt / glücklich; einsam / traurig / unsicher; ängstlich / niedergeschlagen / schuldig / deprimiert / misstrauisch / gereizt

At the moment I feel
cheerful / content / energetic / enthusiastic / relaxed / happy;
lonely, sad, insecure, anxious, depressed, low-spirited, guilty, distrustful, irritable

Wadle LM, Ebner-Priemer UW, Foo JC, et al. Speech Features as Predictors of Momentary Depression Severity in Patients With Depressive Disorder Undergoing Sleep Deprivation Therapy: Ambulatory Assessment Pilot Study. JMIR Mental Health. 2024;11:e49222. doi:10.2196/49222

Textbox S5. Additional eGeMAPS features included in exploratory analysis

spectralFlux_sma3_amean, spectralFlux_sma3_stddevNorm, spectralFluxUV_sma3nz_amean, spectralFluxV_sma3nz_amean, spectralFluxV_sma3nz_stddevNorm, mfcc1_sma3_amean, mfcc1_sma3_stddevNorm, mfcc2_sma3_amean, mfcc2_sma3_stddevNorm, mfcc3_sma3_amean, mfcc3_sma3_stddevNorm, mfcc4_sma3_amean, mfcc4_sma3_stddevNorm, mfcc1V_sma3nz_amean, mfcc1V_sma3nz_stddevNorm, mfcc2V_sma3nz_amean, mfcc2V_sma3nz_stddevNorm, mfcc3V_sma3nz_amean, mfcc3V_sma3nz_stddevNorm, mfcc4V_sma3nz_amean, mfcc4V_sma3nz_stddevNorm, equivalentSoundLevel_dBp, F2bandwidth_sma3nz_amean¸ F2bandwidth_sma3nz_stddevNorm, F3bandwidth_sma3nz_amean, F3bandwidth_sma3nz_stddevNorm

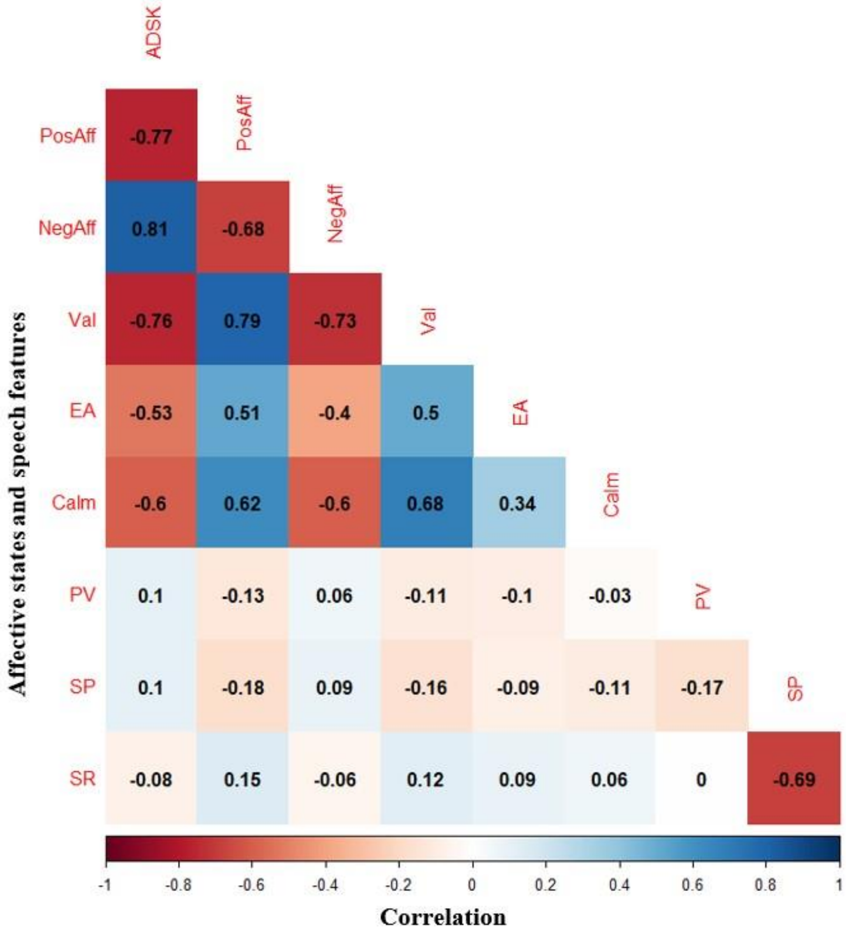

Figure S1. Pearson correlations with person centered variables of the relationship between affective scores and speech features (n between 698 and 716). PosAff: positive affect; NegAff: negative affect, Val: valence; EA: energetic arousal; Calm: calmness; PV: pitch variability; SP: speech pauses; SR: speech rate

Table S1. Multilevel linear regression analysis to predict momentary depression severity and affective states: fixed effects of pitch variability, speech pauses, speech rate, time and time².

Outcome	Fixed				
Predictor	Beta Coefficient	Standardized Beta Coefficient	Standard Error	T	*P*-Value
**ADS-K**					
Intercept	1.27		0.10	12.87	<.001
Time	<0.01		<0.01	0.40	.69
Time-squared	<0.01		<0.01	-0.09	.93
Pitch Variability	0.88	0.14	0.32	2.73	.007
**Positive affect**					
Intercept	2.10		0.13	16.78	<.001
Time	<-0.01		<0.01	-0.97	.33
Time-squared	<0.01		<0.01	-0.13	.90
Pitch Variability	-1.50	-0.18	0.42	-3.56	<.001
**Negative affect**					
Intercept	2.45		0.16	14.86	<.001
Time	<0.01	0.04	<0.01	1.74	.08
Time-squared	<0.01		<0.01	-1.43	.15
Pitch Variability	0.85	0.08	0.43	1.95	.052
**Valence**					
Intercept	43.72		2.70	16.21	<.001
Time	<0.01		<0.01	1.23	.22
Time-squared	<0.01	<0.01	<0.01	1.67	.098
Pitch Variability	-36.50	-0.16	13.61	-2.68	<.008
**Energetic arousal**					
Intercept	42.82		2.71	15.79	<.001
Time	<-0.01	0.11	<0.01	-3.46	<.001
Time-squared	<0.01	<0.01	<0.01	-4.41	<.001
Pitch Variability	-33.21	-0.15	12.48	-2.66	<.001

Wadle LM, Ebner-Priemer UW, Foo JC, et al. Speech Features as Predictors of Momentary Depression Severity in Patients With Depressive Disorder Undergoing Sleep Deprivation Therapy: Ambulatory Assessment Pilot Study. JMIR Mental Health. 2024;11:e49222. doi:10.2196/49222

**Calmness**					
Intercept	40.97		.3.39	12.08	<.001
Time	<0.01		<0.01	0.20	.84
Time-squared	<0.01	<0.01	<0.01	2.49	.01
Pitch Variability	-11.52	-0.05	12.82	-.90	.37
**ADS-K**					
Intercept	1.27		0.10	12.99	<.001
Time	<0.01		<0.01	0.26	.79
Time-squared	<0.01		<0.01	-0.48	.63
Speech Pauses	0.52	0.10	0.18	2.80	.005
**Positive affect**					
Intercept	2.09		0.13	16.64	<.001
Time	<-0.01		<0.01	-0.90	.37
Time-squared	<0.01		<0.01	0.61	.54
Speech Pauses	-1.16	-0.17	0.24	-4.84	<.001
**Negative affect**					
Intercept	2.46		0.16	14.95	<.001
Time	<0.01	0.04	<0.01	1.75	.08
Time-squared	<0.01	<0.01	<0.01	-1.90	.06
Speech Pauses	0.76	0.09	0.25	3.05	.002
**Valence**					
Intercept	43.26		2.69	16.06	<.001
Time	<0.01		<0.01	1.28	.20
Time-squared	<0.01	<0.01	<0.01	2.22	.03
Speech Pauses	-34.06	-0.19	7.71	-4.42	<.001
**Energetic arousal**					
Intercept	42.71		2.71	15.74	<.001
Time	<-0.01	0.11	<0.01	-3.25	.001
Time-squared	<0.01	<0.01	<0.01	-4.17	<.001
Speech Pauses	-14.06	-0.08	7.14	-1.97	.049

**Calmness**					
Intercept	40.58		3.39	11.98	<.001
Time	<0.01		<0.01	0.06	.95
Time-squared	<0.01	<0.01	<0.01	2.98	.003
Speech Pauses	-24.27	-0.12	7.27	-3.34	<.001
**ADS-K**					
Intercept	1.27		0.10	12.93	<.001
Time	<0.01		<0.01	0.26	.80
Time-squared	<0.01		<0.01	-0.22	.83
Speech Rate	-0.11	-0.10	0.05	-2.27	.02
**Positive affect**					
Intercept	2.10		0.13	16.71	<.001
Time	<-0.01		<0.01	-0.91	.36
Time-squared	<0.01		<0.01	0.18	.86
Speech Rate	0.26	0.18	0.06	4.09	<.001
**Negative affect**					
Intercept	2.45		0.16	14.88	<.001
Time	<0.01	0.04	<0.01	1.69	.09
Time-squared	<0.01		<0.01	-1.57	.12
Speech Rate	-0.13	-0.08	0.07	-2.05	.04
**Valence**					
Intercept	43.56		2.70	16.13	<.001
Time	<0.01		<0.01	1.28	.20
Time-squared	<0.01	<0.01	<0.01	1.85	.07
Speech Rate	6.49	0.17	2.03	3.20	.001
**Energetic arousal**					
Intercept	42.77		2.71	15.76	<.001
Time	<-0.01	0.11	<0.01	-3.32	<.001
Time-squared	<0.01	<0.01	<0.01	-4.29	<.001
Speech Rate	4.13	0.11	1.87	2.22	.027

Wadle LM, Ebner-Priemer UW, Foo JC, et al. Speech Features as Predictors of Momentary Depression Severity in Patients With Depressive Disorder Undergoing Sleep Deprivation Therapy: Ambulatory Assessment Pilot Study. JMIR Mental Health. 2024;11:e49222. doi:10.2196/49222

**Calmness**					
Intercept	40.86		3.39	12.05	<.001
Time	<0.01		<0.01	0.14	.89
Time-squared	<0.01	<0.01	<0.01	2.63	.009
Speech Rate	3.43	0.09	1.91	1.80	.07

Table S2. Multilevel linear regression analysis to predict momentary depression severity and affecive: fixed effects of equivalent sound level, spectral flux, spectral flux of voiced regions only and time and time²

Outcome			Fixed		
Predictor	Beta Coefficient	Standardized Beta Coefficient	Standard Error	T	*P*-Value
**ADS-K**					
Intercept	1.29		0.10	13.11	<.001
Time	<0.01		<0.01	0.53	.60
Time-squared	<0.01		<0.01	-1.06	.29
Equivalent sound level	-0.03	-0.30	<0.01	-5.83	<.001
**Positive affect**					
Intercept	2.08		0.13	16.59	<.001
Time	<-0.01		<0.01	-1.06	.29
Time-squared	<0.01		<0.01	0.96	.34
Equivalent sound level	0.05	0.34	<0.01	6.53	<.001
**Negative affect**					
Intercept	2.47		0.16	15.02	<.001
Time	<0.01		<0.01	1.92	.06
Time-squared	<0.01		<0.01	-2.27	.02
Equivalent sound level	-0.04	0.21	<0.01	-4.81	<.001
**Valence**					
Intercept	43.12		2.70	16.01	<.001
Time	<0.01		<0.01	1.32	.19

Time-squared	<0.01		<0.01	2.38	.02
Equivalent sound level	1.09	0.29	0.23	4.63	<.001
**Energetic arousal**					
Intercept	42.31		2.71	15.64	<.001
Time	<-0.01		<0.01	-3.43	<.001
Time-squared	<0.01		<0.01	-3.66	<.001
Equivalent sound level	0.95	0.26	0.22	4.43	<.001
**Calmness**					
Intercept	40.48		3.39	11.95	<.001
Time	<0.01		<0.01	0.09	.93
Time-squared	<0.01		<0.01	3.09	.002
Equivalent sound level	0.76	0.19	0.22	3.50	<.001
**ADS-K**					
Intercept	1.28		0.10	13.02	<.001
Time	<0.01		<0.01	0.09	.93
Time-squared	<0.01		<0.01	-0.71	.48
Spectral Flux	-0.84	-0.22	0.17	-4.81	<.001
**Positive affect**					
Intercept	2.09		0.13	16.64	<.001
Time	<-0.01		<0.01	-0.57	.57
Time-squared	<0.01		<0.01	0.71	.48
Spectral Flux	1.42	0.28	0.23	6.28	<.001
**Negative affect**					
Intercept	2.46		0.16	14.95	<.001
Time	<0.01		<0.01	1.56	.12
Time-squared	<0.01		<0.01	-1.99	.047
Spectral Flux	-0.96	-0.15	0.25	-4.05	<.001
**Valence**					
Intercept	43.33		2.70	16.07	<.001
Time	<0.01		<0.01	1.61	.11
Time-squared	<0.01		<0.01	2.14	.03
Spectral Flux	29.46	0.21	7.23	4.04	<.001

Wadle LM, Ebner-Priemer UW, Foo JC, et al. Speech Features as Predictors of Momentary Depression Severity in Patients With Depressive Disorder Undergoing Sleep Deprivation Therapy: Ambulatory Assessment Pilot Study. JMIR Mental Health. 2024;11:e49222. doi:10.2196/49222

**Energetic arousal**					
Intercept	42.53		2.71	15.70	<.001
Time	<-0.01		<0.01	-3.14	.002
Time-squared	<0.01		<0.01	-3.96	<.001
Spectral Flux	23.56	0.17	6.71	3.51	<.001
**Calmness**					
Intercept	40.58		3.39	11.98	<.001
Time	<0.01		<0.01	0.31	.75
Time-squared	<0.01		<0.01	2.99	.003
Spectral Flux	23.81	0.27	6.86	3.47	<.001
**ADS-K**					
Intercept	1.28		0.10	12.98	<.001
Time	<0.01		<0.01	0.07	.94
Time-squared	<0.01		<0.01	-0.58	.56
Spectral flux of voiced regions only	-0.55	-0.23	0.11	-4.81	<.001
**Positive affect**					
Intercept	2.09		0.13	16.67	<.001
Time	<-0.01		<0.01	-0.54	.59
Time-squared	<0.01		<0.01	0.50	.62
Spectral flux of voiced regions only	0.89	0.28	0.15	5.94	<.001
**Negative affect**					
Intercept	2.46		0.16	14.92	<.001
Time	<0.01		<0.01	1.53	.13
Time-squared	<0.01		<0.01	-1.82	.07
Spectral flux of voiced regions only	-0.55	-0.15	0.16	-3.57	<.001
**Valence**					
Intercept	43.47		2.70	16.09	<.001
Time	<0.01		<0.01	1.63	.10
Time-squared	<0.01		<0.01	1.98	.048
Spectral flux of voiced regions only	16.91	0.20	4.80	3.52	<.001

Wadle LM, Ebner-Priemer UW, Foo JC, et al. Speech Features as Predictors of Momentary Depression Severity in Patients With Depressive Disorder Undergoing Sleep Deprivation Therapy: Ambulatory Assessment Pilot Study. JMIR Mental Health. 2024;11:e49222. doi:10.2196/49222

**Energetic arousal**					
Intercept	42.57		2.71	15.70	<.001
Time	<-0.01		<0.01	-3.13	.002
Time-squared	<0.01		<0.01	-4.03	<.001
Spectral flux of voiced regions only	16.45	0.20	4.40	3.74	.027
**Calmness**					
Intercept	40.68		3.39	12.00	<.001
Time	<0.01		<0.01	0.33	.74
Time-squared	<0.01		<0.01	2.87	.004
Spectral flux of voiced regions only	14.23	0.16	4.51	3.16	.002
